# Health access to immigrants: identifying gaps for social protection in health

**DOI:** 10.11606/S1518-8787.2020054001501

**Published:** 2020-02-12

**Authors:** Baltica Cabieses, Marcela Oyarte

**Affiliations:** I Universidad del Desarrollo Facultad de Medicina Clínica Alemana Instituto de Ciencias e Innovación en Medicina Santiago Chile Universidad del Desarrollo, Facultad de Medicina Clínica Alemana, Instituto de Ciencias e Innovación en Medicina. Santiago, región metropolitana, Chile; II Instituto de Salud Pública de Chile Departamento de Asuntos Científicos Subdepartamento de estudios y evaluación de proyectos Santiago Chile Instituto de Salud Pública de Chile, Departamento de Asuntos Científicos, Subdepartamento de estudios y evaluación de proyectos. Santiago, región metropolitana, Chile

**Keywords:** Emigrants and Immigrants, Health Services Accessibility, Socioeconomic Factors, Health Status Disparities, Health Surveys, Chile

## Abstract

**OBJECTIVE:**

To compare the access to and effective use of health services available among international migrants and Chileans.

**METHODS:**

Secondary analysis of the National Socioeconomic Characterization Survey (CASEN – *Caracterización Socioeconómica Nacional* ), version 2017. Indicators of access to the health system (having health insurance) and effective use of health services (perceived need, appointment or coverage, barriers and need satisfaction) were described in immigrants and local population, self-reported. Gaps by immigrant status were estimated using logistic regressions, with complex samples.

**RESULTS:**

Immigrants were 7.5 times more likely to have no health insurance than local residents. Immigrants presented less perceived need than local residents, together with a greater lack of appointments (OR: 1.7 95%CI: 1.2–2.5), coverage (OR: 2.7 95%CI: 2.0–3.7) and unsatisfied need. The difference between immigrants and locals was not statistically significant in barriers to health care access (α = 0.005).

**CONCLUSIONS:**

Disadvantages persist regarding the access to and use of health services by immigrants as opposed to Chileans compared with information from previous years. It is necessary to reduce the gaps between immigrants and people born in Chile, especially in terms of health system access. This is the first barrier to effective use of services. The generation of concrete strategies and health policies that consider an approach of social participation of the immigrant community is suggested to bring the health system closer to this population.

## INTRODUCTION

The right to health is a universal and inalienable human right. It was recognized globally by most countries in the treaty adopted by the United Nations General Assembly in 1966 and came into force in 1976. In 2002, Latin American countries and the Caribbean agreed to initiate efforts to extend social health protection. It also appears as one of the eight Areas of Action defined in the Health Agenda for the Americas 2008–2017. In this agenda, all the Ministers of Health from Ibero-America committed themselves to combating health exclusion and building integrated social protection systems with the signing of the Iquique Declaration in July 2007^1^ .

Social health protection is a concrete safeguard measure of the human right to health. It is developed through three main and complementary dimensions: (i) horizontal coverage (health system access); (ii) vertical coverage (access to benefits); and (iii) financial protection^2^ . Each dimension is essential for a State to provide and guarantee the right to health in its fundamental aspects. Vertical coverage includes the indicator of effective use of health services or benefits^3 - 5^ , of special relevance for the identification of gaps in health access by the general population and vulnerable groups^6 , 7^ . Differences in the effective use of health benefits between social groups, in the face of the same perceived need, correspond to unjust and preventable differences (social inequities in health) that require constant attention and reparation. The indicator of effective use of health services or benefits can be disaggregated into the chain of health need and demand: (i) perceived health need, (ii) expressed and unexpressed demand, (iii) satisfied and unsatisfied demand^8^ .

In Chile, there is still a debate about repositioning social health protection as a human right for the entire population residing in our territory, regardless of their sex, ethnicity, socioeconomic level or migratory status^9^ . This country has a mixed health system, with a public component (close to 70.0% of the population) that protects the sickest and the poorest, a private component (close to 25.0% of the population) that protects the youngest and wealthiest, and a minor component of the armed forces and public order (5.0%). The Constitution of Chile indicates that free and equal access to health should be provided, but this premise has failed by demonstrating profound health inequalities among social groups, to the detriment of the least advantaged in their socioeconomic position. International migrant population has the right to use the public or private health system if they have a valid residence visa, selecting the type of provision according to co-payment capacity. For migrants going through a visa application process, an opportunity for health access on an equal footing with other migrants and Chileans was created if they document a situation of lack of resources to a witness of faith, usually a social worker, in the same health system (Decree 67, in force since June 2016).

Health protection for international migrants is a global concern. In ancient times and in modern life, moving from one place to another has always offered the opportunity to improve well-being. International migration represents an issue of global attention, reflecting international processes of social inequality and development, conflict and international stratification of labor, and processes of population ageing, to name only some of its dimensions. Added to this, in the globalization era, and given the ease access to information, the advance in communications and the reduced time and cost of moving from one place to another is faster and less costly today than in the past^10^ . Human displacement in all its forms is one of humanity’s greatest challenges. Migration is also recognized as a social determinant of health in the world. The process of migration itself, as well as factors associated with it when some degree of vulnerability or lack of protection of universal rights is experienced, have the potential to affect the physical, mental and emotional health of migrants and their families^10 - 12^ .

The health of migrants is affected when they are not adequately protected. Accidents, hypothermia, burns, cardiovascular accidents, complications in pregnancy and childbirth, diabetes and hypertension are the conditions most frequently mentioned by the World Health Organization^10^ . On the other hand, risks associated with displacement, such as psychosocial disorders, reproductive health problems, increased neonatal mortality, drug abuse, nutritional disorders, alcoholism and exposure to violence have also been described^10 , 11 , 12^ . One of the aspects of growing interest on a global scale concerns the continuity of care in the transnational setting. This is due to the risks of health care interruption, mainly due to lack of health access in the receiving country^10 , 11 , 12^ .

The most recent estimate from the Department of Foreigners and Migration of the Ministry of Interior and Public Security in Chile, from April 2018, indicated there would be a 6.6% international migrant population, corresponding to a marked increase from 2012 census statistics that estimated 2.5-3.0%. The majority of international migrants come from countries of the Southern Cone (called “south-south” migration pattern typical of the Latin American region), especially Argentina, Colombia, Venezuela, Ecuador, Bolivia and Haiti (2017 Short Census). It is important to recognize the great socioeconomic variability that the international migrant population presents in Chile and in the world. Just as there are migrants of high social hierarchy, there are also those of medium and low socioeconomic levels^11 , 12^ . The migratory experience can have a negative impact on all social strata. However, health problems tend to concentrate on those migrants who experience poverty, exclusion and discrimination^13 , 14^ .

In both Chile and the region as a whole, the evidence for the use of health services by international migrants is limited. Studies conducted by Cabieses et al.^11 - 15^ reported that the immigrant population would underutilize healthy child control compared with the local population, would use Pap smears screening similarly to the national one, and would use more prenatal and gynecological care services than locals. In addition, a clear socioeconomic gradient is observed in most of these services.

The aim of this study was to compare the effective use of the health services available among international and Chilean migrants. This comparison enables us to identify inequality gaps in the use of services, which are potentially modifiable, can have a negative impact on the health of migrants and run counter to the universal goal of social health protection.

## METHODS

Cross-sectional observational study. Secondary analysis of the National Socioeconomic Characterization Survey (CASEN – Caracterización Socioeconómica Nacional), version 2017^16^ . This survey allows us to observe general patterns of the population that self-reports as an immigrant (born abroad) and to compare it with the Chilean population (born in Chile).

The CASEN survey is developed periodically by the Ministry of Social Development. It is an instrument for diagnosis, evaluation and targeting, with the objective of knowing the socioeconomic conditions of the country’s households, especially those groups defined as priorities by social policies. This anonymous and free survey uses a complex probabilistic sample, allowing the representation of individuals in private residences of 324 towns of the 16 regions of the country, excluding towns with difficult access and institutionalized people^16^ .

CASEN had 70,948 households in 2017, considering a non-response rate of 26.6%. This survey contains information of 216,439 people residing in private households, of which 207,603 (representing 16,843,471) were Chilean and 6,811 (representing 777,407) immigrants. Thus, 94.6% of the population represented was Chilean and 4.4%, immigrant; 0.94% of the individuals did not report their migratory status, and therefore were excluded from the analysis. Data collection was carried out by a structured direct interview with habitual residents or an adult of the family, able to answer for the other members of the household.

The study variables were:

1.Access to the health system

- Affiliation of a health insurance system [yes/no].
- Type of health insurance [public/private/other].

Both were created by recoding the question “Which health insurance system do you use? [1. Public System FONASA group A, 2. Public System, FONASA group B, 3. Public System FONASA group C, 4. Public System, FONASA group D, 5. Public System FONASA does not know the Group, 6. FF. AA. and Order, 7. ISAPRE, 8. None (private), 9. Other system].”

**Figure 1:**
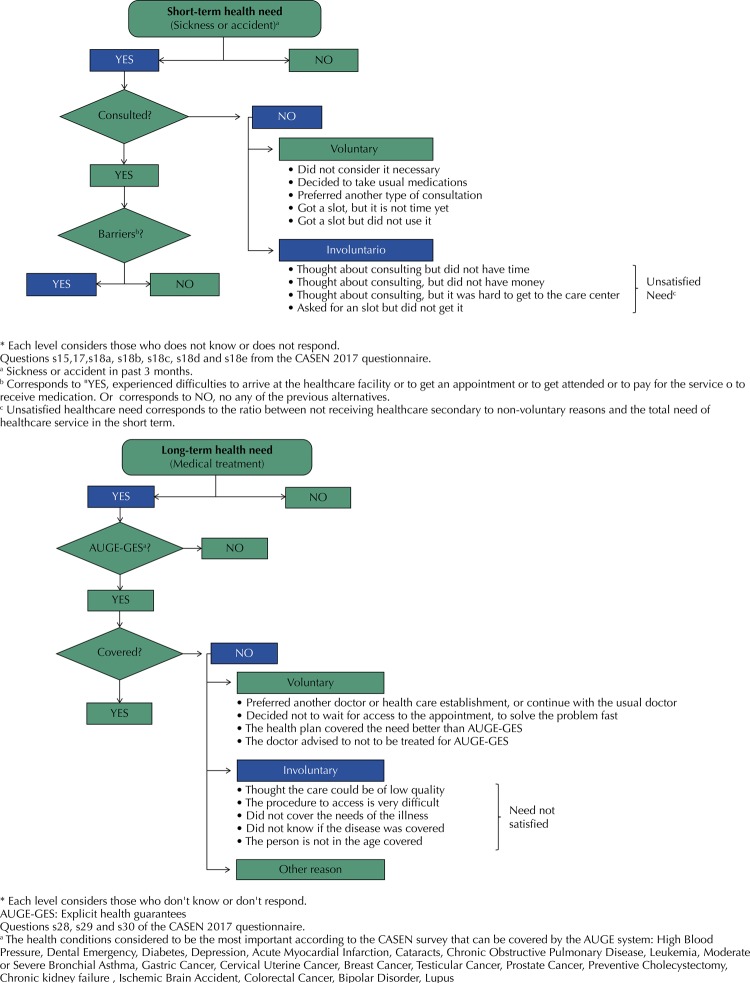
Effective use of health services for short and long-term needs: perceived need, expression of demand, satisfaction of perceived need, coverage of Explicit Health Guarantees (AUGE-GES) and barriers to access to health care. National Socioeconomic Characterization Survey (CASEN), Chile, 2017.

2.Indicators of effective use of health services17: Self-reports constructed from the variables available in the CASEN 201716 survey ( Figure 1 ).

- Perceived need for health: Short-term (Did you have any health problems or accidents in the last three months? [yes/no]) and long-term (Have you been receiving medical treatment for the past 12 months? [yes/no]).
- Demand expressed and not expressed: have consulted the Chilean health system, or not, for those who reported having had some short-term health need [yes/no]; and to be covered or not by the AUGE-GES system in the case of long-term needs.
- Satisfied and unsatisfied demand: (yes) Appointment or coverage of the perceived health need in short or long term, respectively, or no appointment or coverage for voluntary reasons. (No) no appointment or coverage for involuntary reasons.
- Reasons for not having an appointment (Why have you not had an appointment or health care?) or coverage (Why was this medical treatment not covered by the AUGE-GES system?).
- Barriers to health care: Presence of problems during the appointment due to short-term need [yes/no]. Indicator constructed from the questions: When you had an appointment, did you have any of the following problems? a) arriving for the appointment, hospital, clinic, etc. b) to get an appointment/health care (slot). c) to be attended at the establishment (delay in care, changes in the slot, etc.) d) paying for care due to cost e) delivery of medications at the healthcare establishment or access to them because of the costs.

In cases of short and long-term perceived health need, a higher indicator value reflected a worse health condition. On the other hand, a higher value in the indicators: no access to a health insurance system, demand not expressed, no appointment, no coverage, no appointment or no coverage for involuntary reasons, unsatisfied need and presence of barriers to access, indicated a worse access to health services.

3.Sociodemographic control variables: age, sex, area of residence [urban/rural], educational level [no education/basic/high/technical or professional], activity status [employed/unemployed/active], autonomous quintile of total household income.

The indicators of access to the health system and effective use of health services were analyzed descriptively for immigrants and people born in Chile, in totality and stratified according to sociodemographic variables. The independence between migratory status and indicators of effective use of health services and access to the health system was analyzed through Pearson’s test χ^2^ with second-order Rao-Scott correction (statistic F).

Logistic regression models were performed, adjusted for sociodemographic variables, considering the use of a health insurance system, Demand satisfied in the short and long-term and Barriers to care, as dependent variables, and immigrant status as an independent variable. This is for the total population and for people over 14 years old (adults), additionally considering the activity condition variable.

Database management and data analysis was performed using STATA 14 (StataCorp) software, considering the complex design of the survey (conglomerate stratum and expansion factor) and an estimate of variance by linearization by Taylor series. A significance of 0.05 and 95% confidence was considered for all analyses.

## RESULTS

The total of 16.3% of the country’s immigrants affirmed that they did not use a health insurance system, a value seven times higher compared with the Chileans (2.3%).

Immigrants showed significantly less perceived health need than those born in Chile in both short and long-term. In the short-term, in immigrants, 15.1% of the total population reported illness or accident in the three months prior to the survey (vs. 20.2% in Chileans); of these, 9.3% did not have an appointment and 1.7 of every 100 people did not meet their health need by not having an appointment for reasons beyond their control. For Chileans, these values will correspond to 6.1% and 0.7 out of 100, respectively, significantly lower than in immigrants. On the contrary, immigrants and Chileans reported a similar proportion of problems during the appointment (access barriers): approximately 25.0% of consultants for short-term needs (25,179 immigrants, 774,792 Chileans). In the long term, 9.6% of the immigrants were in medical treatment for some illness during the year prior to the survey (vs. 26.2% in Chileans), of which 44.4% were for another health condition different from the 20 main AUGE-GES pathologies treated. This percentage corresponded to 27.2% in Chileans. Immigrants significantly reported a higher proportion of no coverage (44.6% immigrants, 15.2% Chileans) and unsatisfied needs (15.0% immigrants, 3.2% Chileans), being specifically 2.9 and 4.7 times higher in immigrants than in Chileans, respectively ( Table 1 ). In terms of Odds Ratio (OR), the results were consistent with what was observed at the descriptive level. However, there are some differences in the magnitude of the disparities between immigrants and locals after adjusting for sociodemographic variables.

**Table 1:** Access to the health system, effective use of health services for short and long-term needs, and barriers to access to health care for immigrants and those born in Chile. National Socioeconomic Characterization Survey (CASEN), Chile, 2017

	Immigrants		Chileans		Ratio Immigrant: Chilean	
	
	Quantity	Indicator	Quantity	Indicator		
**No health insurance**	123.013	**16.3**	378.239	**2.3**	7.1	*

**Perceived need (Short-term)**	116.187	**15.1**	3.361.433	**20.2**	0.8	*
**Unexpressed demand**	10.720	**9.3**	203.530	**6.1**	1.5	*
Reasons for missing the appointment:						
Voluntary	8.042	**80.9**	158.604	**87.1**	0.9	
Involuntary	1.904	**19.1**	23.449	**12.9**	1.5	
**Unsatisfied need**	1.904	**1.7**	23.449	**0.7**	2.4	*
**Access barriers**	25.179	**24.7**	774.792	**25.4**	1.0	
Problems to:						
Arrive for the appointment	4.105	**4.0**	218.147	**7.1**	0.6	*
Get a slot/care	14.724	**14.4**	390.782	**12.8**	1.1	
To be attended in the establishment	13.186	**13.0**	501.580	**16.4**	0.8	
Paying for care	5.585	**5.5**	171.711	**5.6**	1.0	
Delivery of medicines	7.403	**7.5**	227.843	**7.2**	1.0	

**Perceived need (Long- term)**	74.216	**9.6**	4.374.959	**26.2**	0.4	*
**No covered**	16.878	**44.6**	450.398	**15.2**	2.9	*
Reasons for the lack of appointment:						
Voluntary	6.974	**44.2**	230.410	**55.3**	0.8	
Another reason, not specified	3.865	**24.5**	96.017	**23.0**	1.1	
Involuntary	4.953	**31.4**	90.330	**21.7**	1.4	
**No satisfied need**	4.953	**15.0**	90.330	**3.2**	4.7	*

* Indicator not independent of immigrant status (α=0.05). Test F, second order correction Rao and Scott.

After adjusting for socio-demographic variables, immigrants have 7.5 times more chances of not having health insurance than Chileans. The odds were 6.2 times greater in immigrants than in Chileans over 15 years old and considering the activity condition (employed, unemployed and inactive).

Sex, having secondary or higher education compared with no schooling, and belonging to the richest income quintile were significant factors in the lack of health provision, disadvantageous to women, people with no schooling and lower income for the total population. In people over 15 years old, in addition, age and unemployment in reference to occupation will turn out to be significant. On the other hand, having a high school education in relation to no schooling ceased to be significant ( Table 2 ).

**Table 2:** Determinants of lack of health provision, demand not expressed by short-term needs, needs not satisfied in the short- term, presence of access obstacles during care, not covered Explicit Health Guarantees (AUGE-GES) for long-term needs and needs not satisfied in long-term. National Socioeconomic Characterization Survey (CASEN), Chile, 2017.

	Access to the health system				

	No health insurance				

	OR	95CI%			
Model 1					
**Chilean**	**7.5**	**6.08**	**-**	**9.29**	*
**Immigrant**	**1**				
Rural	1.1	0.92	-	1.21	
Urban	1				
Woman	0.6	0.56	-	0.66	*
Male	1				
Age	1.0	0.99	-	1.00	
Basic	1.2	0.83	-	1.70	
Medium	1.8	1.25	-	2.69	*
Superior	2.6	1.82	-	3.64	*
Without schooling	1				
Quintile II	1.1	0.92	-	1.23	
Quintile III	1.0	0.91	-	1.22	
Quintile IV	1.0	0.78	-	1.29	
Quintile V (richer)	0.6	0.50	-	0.78	*
Quintile I (poorest)	1				
cte.	0.0	0.02	-	0.03	*

Model 2					
**Chilean**	**6.2**	**5.10**	**-**	**7.43**	*
**Immigrant**	**1**				
Rural	1.0	0.91	-	1.20	
Urban	1				
Woman	0.6	0.52	-	0.64	*
Male	1				
Age	1.0	0.98	-	0.99	*
Basic	1.2	0.89	-	1.68	
Medium	1.3	0.93	-	1.79	
Superior	1.7	1.20	-	2.38	*
Without schooling	1				
Quintile II	1.1	0.93	-	1.24	
Quintile III	1.1	0.97	-	1.31	
Quintile IV	1.1	0.92	-	1.33	
Quintile V (richer)	0.8	0.62	-	0.98	*
Quintile I (poorest)	1				
Unemployed	2.9	2.48	-	3.30	*
Inactive	1.0	0.90	-	1.11	
Employed	1				
cte.	0.0	0.03	-	0.05	*

*Significant, with a significance of 0.05

Model 1: N=17,177,754 Df=1,295 F=96.05

Model 2: N=13,857,106 (15 years or more) Df=1,295 F=83.76

Among the 632,770 immigrants with health insurance, 80.0% used the public system, 18.0% from the private system and 2.0% from the armed forces (FF.AA.) or other, while in Chileans these percentages were 82.1%, 15.0% and 2.9%, respectively. The proportion of men and women using the private health system was higher in both immigrants and Chileans. 15.2% (95%CI:12.26–18.79%) of women with pensions used the public system, while in men, 21.0% (95%CI:16.78–26.03%) were immigrants. [Results not presented in the table]

The immigrant population presented 21.0% (OR = 0.79) less odds of having short-term health needs (illnesses or accidents) than Chileans. However, their odds of not getting an appointment were 1.7 times higher because of this need and the odds of not satisfying this need or not getting an appointment for involuntary reasons were 3.1 higher. All this after adjusting for sociodemographic variables. This situation was maintained for people older than 15 years. Sex, age, schooling and belonging to the richest quintile were significant variables in non-expression of demand. Education ceased to be significant in individuals over 15 years old, and the rest of the income quintiles (with the exception of the III) and the activity condition became significant ( Table 3 ).

**Table 3:** Determinants of demand not expressed by short-term needs, unsatisfied needs in the short and long-term, presence of barriers to access during care and no coverage Explicit Health Guarantees (AUGE-GES). National Socio-economic Characterization Survey (CASEN), Chile, 2017.

	Short–term									Long–term					
	
	Not expressed demand^a^			Unsatisfied need^b^			Access obstacles^c^			No coverage AUGE–GES^a^			Unsatisfied need^b^		
				
	OR	95CI%		OR	95CI%		OR	95CI%		OR	95CI%		OR	95CI%	
**Chilean**	**1.7**	**1.22–2.47**	*	**3.1**	**1.31–7.44**	*	**1.2**	**0.75–1.84**		**2.7**	**1.97–3.73**	*	**3.3**	**1,89–5,74**	*
**Immigrant**	**1**			**1**			**1**			**1**					
Rural	1.2	0.94–1.43		1.2	0.71–1.88		1.0	0.86–1.18		0.6	0.50–0.68	*	0.6	0.46–0.86	*
Urban	1			1			1			1					
Woman	0.8	0.72–0.88	*	0.9	0.66–1.28		1.0	0.96–1.08		1.0	0.88–1.04		1.0	0.89–1.23	
Male	1			1			1			1					
Age	1.0	1.00–1.01	*	1.0	1.01–1.02	*	1.0	1.00–1.01		1.0	0.98–0.99	*	1.0	0.98–0.99	*
Basic	1.5	1.14–1.92	*	1.8	0.83–4.08		1.0	0.90–1.13		1.0	0.83–1.33		0.9	0.61–1.26	
Medium	1.8	1.38–2.25	*	2.3	1.08–4.92	*	1.0	0.87–1.11		1.7	1.35–2.15	*	1.2	0.83–1.73	
Superior	1.8	1.39–2.35	*	2.0	0.86–4.62		0.9	0.80–1.04		3.4	2.63–4.29	*	2.0	1.40–3.00	*
No schooling	1			1			1			1					
Quintile II	0.9	0.74–1.11		0.8	0.47–1.22		0.9	0.81–0.99	*	1.3	1.08–1.53	*	1	0.75–1.35	
Quintile III	1.0	0.78–1.18		0.7	0.41–1.06		0.8	0.69–0.90	*	1.8	1.52–2.08	*	1.6	1.19–2.26	*
Quintile IV	0.9	0.69–1.08		0.9	0.47–1.93		0.7	0.58–0.74	*	2.7	2.30–3.22	*	1.9	1.43–2.63	*
Quintile V (richer)	0.6	0.44–0.74	*	0.3	0.14–0.69	*	0.4	0.35–0.50	*	5.5	4.57–6.53	*	2.3	1.69–3.27	*
Quintile I (poorest)	1			1			1			1					
cte.	0.0	0.03–0.05	*	0.0	0.00–0.00	*	0.4	0.32–0.43	*	0.1	0.09–0.16	*	0.0	0.03–0.07	*

**Chilean**	**1.7**	**1.18–2.40**	*	**2.89**	**1.17–7.13**	*	**1.2**	**0.77–1.88**		**2.8**	**2.01–3.85**	*	**3.3**	**1.87–5.78**	*
**Immigrant**	**1**			**1**			**1**			**1**			**1**		
Rural	1.1	0.89–1.27		1.1	0.64–1.75		1.0	0.83–1.13		0.6	0.52–0.70	*	0.6	0.47–0.89	*
Urban	1			1			1			1			1		
Woman	0.8	0.72–0.89	*	1.0	0.71–1.52		1.0	0.98–1.13		1.0	0.88–1.06		1.1	0.93–1.31	
Male	1			1			1			1			1		
Age	1	1.00–1.01	*	1.0	1.00–1.02		1	1.00–1.01		1.0	0.99–0.99	*	1.0	0.98–0.99	*
Basic	1.0	0.71–1.35		1.2	0.46–2.90		1.1	0.92–1.36		1.1	0.84–1.54		0.8	0.51–1.24	
Medium	1.0	0.74–1.40		1.1	0.44–2.70		1	0.81–1.23		2.0	1.51–2.79	*	1.1	0.70–1.85	
Superior	1.0	0.72–1.37		0.9	0.32–2.41		0.9	0.71–1.11		4.2	3.05–5.71	*	1.9	1.18–3.18	*
No schooling	1			1			1			1			1		
Quintile II	0.8	0.66–0.98	*	0.7	0.42–1.10		0.9	0.82–1.02		1.2	1.05–1.47	*	1.0	0.72–1.34	
Quintile III	0.8	0.69–1.04		0.6	0.37–0.96	*	0.8	0.71–0.95	*	1.7	1.47–2.04	*	1.6	1.18–2.31	*
Quintile IV	0.7	0.59–0.92	*	0.8	0.40–1.48		0.7	0.64–0.82	*	2.5	2.13–3.02	*	1.9	1.39–2.67	*
Quintile V (richer)	0.5	0.39–0.68	*	0.3	0.12–0.62	*	0.5	0.39–0.57	*	5.0	4.20–6.08	*	2.43	1.71–3.44	*
Quintile I (poorest)	1			1			1			1			1		
Unemployed	0.7	0.47–0.93	*	0.8	0.39–1.78		1.6	1.17–2.09	*	1.4	1.06–1.76	*	2.1	1.41–3.01	*
Inactive	0.8	0.67–0.86	*	0.5	0.30–0.76	*	1.0	0.95–1.11		1	0.89–1.11		1	0.81–1.23	
Occupied	1			1			1			1			1		
cte.	0.1	0.07–0.16	*	0.0	0.00–0.02	*	0.3	0.27–0.46	*	0.1	0.05–0.12	*	0.0	0.02–0.07	*

*Significant, with a significance of 0.05

AUGE -GES: Explicit Health Guarantee System

Immigrants had 2.0% (OR = 0.98) times less chance of presenting access barriers than Chileans. After adjusting for socio-demographic variables, this situation was reversed. Immigrants had a greater chance of presenting access barriers both for the entire population and for those aged 15 years or older. It was not significant for none of the cases ( Table 3 ).

For long-term needs (treatment of some diseases), immigrants had greater odds of not having their treatment covered despite having a lesser chance of receiving treatment. Specifically, adjusting for sociodemographic variables, immigrants had 53.0% (OR = 0.47) times less odds of being in treatment for some disease than Chileans and 37.0% (OR = 0.63) times less odds that this disease was an AUGE-GES pathology. Among those with AUGE-GES pathologies, the immigrant population had 2.7 times more odds of not being covered and 3.3 times more odds of not satisfying this necessity for coverage than Chileans. Immigrants older than 15 years had a greater chance of not being covered by AUGE-GES and not satisfying their long-term need compared with Chileans. With the exception of sex and basic education in contrast to no schooling, all variables considered were significant in no coverage and no satisfaction. In people over 15 years old, it was also not significant to have a secondary education in contrast to having no schooling ( Table 3 ).

**Figure 2:**
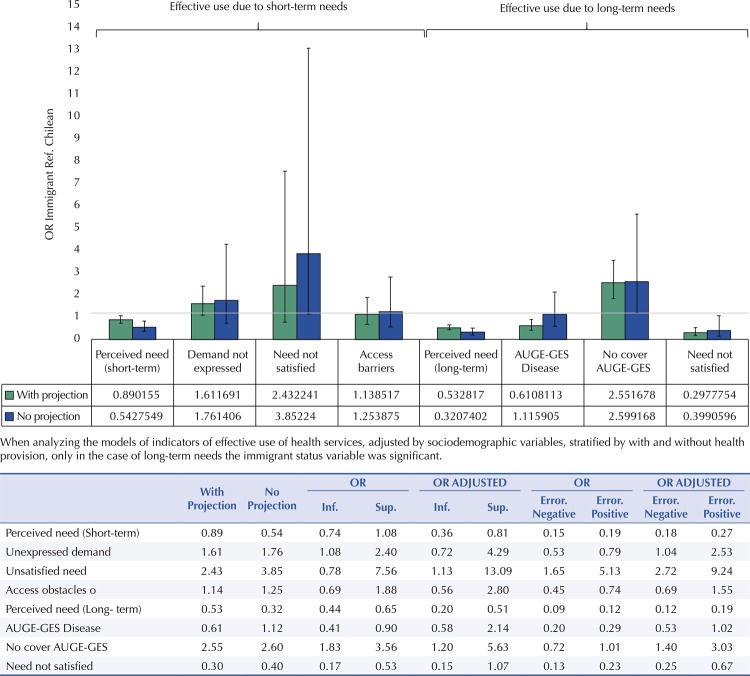
Relative disparity between immigrants and Chileans in terms of access to the health system and effective use of health services for short and long-term needs, expressed as the Odds Ratio (OR) adjusted by socio-demographic variables, according to health forecast. National Socioeconomic Characterization Survey (CASEN), Chile, 2017.

When analyzing the models of indicators of effective use of health services stratified with and without health provision, the presence of long-term needs and coverage of this immigrant status variable was significant ( Figure 2 ).

## DISCUSSION

The results of this study indicate that immigrants are still at a disadvantage in terms of access and use of health services compared with Chileans. Immigrants presented 7.5 times more chances of not having health insurance than Chileans and a lower perceived need but less appointment, coverage or need satisfaction in the short and long-term. Immigrants were 1.7 times more likely to get an appointment in case of an illness or accident and 3.1 times more likely to not satisfy their need by not attending the appointment for reasons out of their control than people born in Chile. On the other hand, the immigrant population were 2.7 times more likely to not have their treatment covered by AUGE-GES and 3.3 times more likely to not satisfy this need for coverage than Chileans.

Many countries have been challenged by the global increase in international migrants recently. This brings a series of challenges for diverse sectors, education, health and social protection, just to name a few, which become more complex to face when this population is in conditions of social vulnerability. Health protection in international migrants is a global concern, and access to health services is a fundamental aspect of this work^17^ .

The percentage of first-generation immigrants present in the country increased 0.7% in Chile between 2013 and 2015, and it represents 110,738 immigrants^18^ . On the other hand, it has been reported that a significant proportion of them live in conditions of social vulnerability. Because they are in an irregular situation, poverty, inadequate housing conditions, unemployment or informal employment and processes of discrimination and abuse or because of the difficulty in obtaining official residence identity document in the country, which, in matters of health access, is essential in order to use the health pension system^19^ . The number of 15.7% immigrants were not affiliated to any health system in 2015, a value 5.8 times greater than that compared with people born in Chile. The immigrant population was at a disadvantage as to the local population in other indicators of health access, such as the medical care rate for health problems and the issue with health care access^18^ .

The same problems are observed in previous years by Cabieses et al. regarding the effective use of health services for short and long-term needs and access to the health system^17^ . Even more worrying is that some of these indicators have increased between 2013 and 2017. 15.6% of immigrants had no health insurance in 2013, while it was 16.3% in 2017; 8.9% of those with short-term needs did not had an appointment because of this need in 2013, while it was 9.4% in 2017. 27.9% of those in treatment for an AUGE-GES disease were not covered by it in 2013, while this value was 44.6% in 2017.

It is necessary to reduce inequality gaps in health access and use of health services by the migrant population. To this end, concrete health strategies and policies are necessary, allowing, by social determinants of health, to protect the health and well-being of migrants, from the appearance of short and long-term health problems, and factors that may favor them, to their effective resolution. Interventions that consider a social participation approach of the international migrant community and that bring the Chilean health system closer to this population are widely recommended^17^ . Even more so in Chile, where according to the CASEN survey (2015), 18.5% of 12 years old or older immigrants participated or participate in some organization or group^18^ . The CASEN survey in all its versions is nationally representative and allows a diagnosis of the situation of the immigrant population in the country with respect to various socioeconomic conditions. In this specific case of health, it provides information about health conditions (needs, general state of health and permanent or long-term conditions), access to the system, health services, health insurance, use of these and associated payments, which in turn may be associated with each other and with a wide range of demographic, cultural and socioeconomic factors^17^ . Despite this, the sample selection process of the survey does not seek to represent the international migrant population in particular and the country of birth (which allows stratification between immigrant and the Chilean) has non-response percentages (responds “does not know”) of 0.9% in 2017. On the other hand, the CASEN survey only allows the analysis of residents in homes, leaving out of the scenario the number of immigrants in street situations, which, according to local media, are increasing^20^ . The analyses carried out from this survey are limited to the variables present in it. Some of the cultural challenges such as differences in perceptions, traditions and lifestyles, among others, represented as barriers to integration and therefore into obstacles and challenges in access to health systems and services^19^ cannot be analyzed directly. It is therefore widely useful in the future to complement the analyses presented in this manuscript.

This study indicates that disadvantages persist in access to health services and their use among immigrants compared with those born in Chile. It is necessary to reduce the gaps between immigrants and Chileans, especially in terms of affiliation to a health system. This is the first barrier to the effective use of services. We suggest the generation of concrete strategies and health policies that consider a social participation approach of the immigrant community and, additionally, bring the health system closer to this population.

Demand model (short-term): N=3,415,253Df=1,289 F=10.64. Model Satisfaction requirement (short-term): N=3,393,049 Df=1,289 F=6.91. Model Barriers: N=3,146. Df=1,288 F=17.78. Adult demand model (short-term): N=2,797,755 Df=1,288 F=6.54. F=6,08. Model Satisfaction of needs in adults (short-term): N=2,778,419 Df=1,288 F=3.72. Model Obstacles in adults: N=2,563,952 Df=1,287 F=13.76. Model Coverage: N=2,974,923 Df=1,295 F=118.55. Model Coverage in adults: N=2,840,844Df=1,295 F=25.94. Model Satisfaction (long-term): N=2,853,809 Df=1,295 F=100.76. Model Adult satisfaction (long-term): N=2,727,317 Df=1,295 F=21.8
