# Supplementary material for: Health access to immigrants: identifying gaps for social protection in health
Source: Rev Saude Publica. 2020 Feb 12;54:20. doi: 10.11606/S1518-8787.2020054001501 (PMC7017981; doi:10.11606/S1518-8787.2020054001501)
Supplement: Translation

# Translation: Acceso a salud en inmigrantes: identificando brechas para la protección social en salud

**Authors:** Baltica Cabieses, Marcela Oyarte

**Affiliations:** I Chile Universidad del Desarrollo, Facultad de Medicina Clínica Alemana, Instituto de Ciencias e Innovación en Medicina. Santiago, región metropolitana, Chile; II Chile Instituto de Salud Pública de Chile, Departamento de Asuntos Científicos, Subdepartamento de estudios y evaluación de proyectos. Santiago, región metropolitana, Chile

**Keywords:** Emigrantes e Inmigrantes, Accesibilidad a los Servicios de Salud, Factores Socioeconómicos, Disparidades en el Estado de Salud, Encuestas Epidemiológicas, Chile

## Abstract

**OBJETIVO:**

Comparar el acceso y uso efectivo de servicios de salud disponibles entre migrantes internacionales y chilenos.

**MÉTODOS:**

Análisis secundario de la encuesta poblacional de Caracterización Socioeconómica Nacional (CASEN), versión 2017. Se describieron indicadores de acceso al sistema de salud (tener previsión de salud) y uso efectivo de servicios de salud (necesidad sentida, consulta o cobertura, barreras y satisfacción de la necesidad) en inmigrantes y locales, autorreportados. Las brechas por condición de inmigrante se estimaron utilizando regresiones logísticas, con muestras complejas.

**RESULTADOS:**

Los inmigrantes presentaron 7,5 veces más chances de no tener previsión de salud que los locales. Los inmigrantes presentaron una menor necesidad sentida que los locales, en conjunto con una mayor falta de consulta (OR: 1,7 IC95%: 1,2–2,5), cobertura (OR: 2,7 IC95%: 2,0–3,7) e insatisfacción de necesidades. La diferencia entre inmigrantes y locales no fue estadísticamente significativa en barreras de acceso a atención en salud (α = 0,005).

**CONCLUSIONES:**

Persisten las desventajas en acceso y uso a servicios de salud en inmigrantes en comparación con los nacidos en Chile en contraste con información de años anteriores. Es necesario reducir las brechas entre inmigrantes y nacidos en Chile, sobre todo en cuanto a pertenencia a un sistema de salud. Esta es la primera barrera para un uso efectivo de servicios. Se sugiere generar estrategias concretas y políticas en salud que consideren un enfoque de participación social de la comunidad inmigrante y, adicionalmente, acerquen al sistema de salud a esta población.

## INTRODUCCIÓN

El derecho a la salud es un derecho humano universal e inalienable. Fue reconocido de forma global por la mayoría de los países del mundo en el tratado adoptado por la Asamblea General de las Naciones Unidas en 1966 y puesto en vigor en 1976. En 2002, países de América Latina y el Caribe acordaron iniciar esfuerzos para extender la protección social en salud. Esta aparece también como una de las ocho Áreas de Acción definidas en la Agenda de Salud de las Américas 2008–2017. En dicha agenda, todos los Ministros de Salud de Iberoamérica se comprometieron a combatir la exclusión en salud y construir sistemas integrados de protección social con la firma de la declaración de Iquique en Julio 2007^1^ .

La protección social en salud es una medida concreta de garantía del derecho humano en salud. Se desarrolla a través de tres dimensiones principales y complementarias entre sí: (i) cobertura horizontal (acceso al sistema de salud); (ii) cobertura vertical (acceso a prestaciones); y (iii) protección financiera^2^ Cada dimensión es esencial para que un Estado otorgue y garantice el derecho a la salud en sus aspectos fundamentales. Dentro de la cobertura vertical está el indicador de uso/utilización efectiva de servicios o prestaciones de salud^3 - 5^ , de especial relevancia para la identificación de brechas de acceso a salud por parte de población general y grupos vulnerables^6 , 7^ . Diferencias de uso efectivo de prestaciones de salud entre grupos sociales, ante igual necesidad sentida, corresponde a diferencias injustas y prevenibles (inequidades sociales en salud) que requieren de constante atención y reparación. El indicador de uso/utilización efectiva de servicios o prestaciones de salud puede desagregarse en la cadena de necesidad y demanda en salud: (i) necesidad sentida de salud, (ii) demanda expresada y no expresada, (iii) demanda satisfecha y no satisfecha^8^ .

En Chile, aún está presente el debate de reposicionar la protección social en salud como un derecho humano para toda la población residente en nuestro territorio, independientemente de su condición de género, etnia, nivel socioeconómico o estatus migratorio^9^ . Este país cuenta con un sistema de salud mixto, con componente público (cerca del 70,0% de la población) que protege a los más enfermos y a los más pobres, componente privado (cerca del 25,0% de la población) que protege a los más jóvenes y adinerados, y componente menor de fuerzas armadas y del orden público (5,0%). La Constitución de Chile indica que se debe brindar acceso libre e igualitario a la salud, pero esta premisa ha fallado al demostrarse profundas desigualdades en salud entre grupos sociales, en desmedro de los menos aventajados en su posición socioeconómica. La población migrante internacional cuenta con derecho a uso del sistema de salud público o privado si tiene su visa de residencia vigente, seleccionando el tipo de previsión según capacidad de copago. Para migrantes con visa en trámite, se creó la oportunidad de acceso a salud en igualdad de condición que los demás migrantes y chilenos si es que documentan situación de carencia de recursos a un testigo de fe, habitualmente trabajador social, en el mismo sistema de salud (Decreto 67, vigente desde junio del 2016).

La protección de la salud por parte de migrantes internacionales es de preocupación global. En tiempos antiguos y en la vida moderna, el desplazamiento de un lugar a otro siempre ha ofrecido la oportunidad de mejor bienestar. La migración internacional representa un tema de atención mundial, al ser reflejo de procesos internacionales de desigualdad social y de desarrollo, de conflicto y de estratificación internacional del trabajo, y de procesos de envejecimiento poblacional, por mencionar algunas de sus dimensiones. Sumado a esto, en la era de la globalización, y dadas las facilidades de acceso a información, el avance en las comunicaciones y el menor tiempo y costo de los traslados, el llegar de un lugar a otro resulta hoy en día más rápido y menos costoso que en el pasado^10^ . El desplazamiento humano en todas sus formas es uno de los mayores desafíos de la humanidad. La migración es además un reconocido determinante social de la salud en el mundo. El proceso de migrar en sí mismo, así como factores asociados a este cuando se experimenta algún grado de vulnerabilidad o falta de protección a derechos universales, tienen el potencial de afectar la salud física, mental y emocional de las personas migrantes y sus familias^10 - 12^ .

La salud de personas migrantes puede verse afectada cuando no cuentan con adecuada protección. Accidentes, hipotermia, quemaduras, accidentes cardiovasculares, complicaciones del embarazo y el parto, diabetes e hipertensión, son las afecciones más mencionadas por la Organización Mundial de la Salud^10^ . Por otra parte, riesgos derivados de los desplazamientos, tales como trastornos psicosociales, problemas de salud reproductiva, mayor mortalidad neonatal, uso indebido de drogas, trastornos nutricionales, alcoholismo y exposición a la violencia, también han sido descritos^10 , 11 , 12^ . Uno de los aspectos de creciente interés a escala global tiene relación con la continuidad del cuidado en el escenario transnacional. Esto por los riesgos en salud de la interrupción de la atención, fundamentalmente por falta de acceso a ella en el país receptor^10 , 11 , 12^ .

La más reciente estimación del Departamento de Extranjería y Migración del Ministerio del Interior y Seguridad Pública en Chile en abril del 2018 indicaba que existiría un 6,6% de población migrante internacional, correspondiendo a un marcado aumento desde estadísticas censales del 2012 que estimaban un 2,5-3,0%. La mayoría de los migrantes internacionales provienen de países del cono sur (el llamado patrón migratorio “sur-sur” propio de la región de Latinoamérica), en especial Argentina, Colombia, Venezuela, Ecuador, Bolivia y Haití (Censo abreviado del 2017). Es relevante reconocer la gran variabilidad socioeconómica que la población migrante internacional presenta en Chile y el mundo. Así como existen migrantes de alta jerarquía social, también los hay de nivel socioeconómico medio y bajo^11 , 12^ . La experiencia migratoria puede impactar negativamente en todos los estratos sociales; sin embargo, los problemas de salud tienden a concentrarse en aquellos migrantes que experimentan pobreza, exclusión y discriminación^13 , 14^ .

Tanto en Chile como en la región en su conjunto, existe evidencia limitada del uso de servicios de salud por parte de población migrante internacional. Estudios realizados por Cabieses y colaboradores^11 - 15^ han reportado que la población inmigrante sub-utilizaría el control de niño sano comparado con la población local, utilizaría en forma similar el *screening* de Papanicolaou a la nacional, y utilizaría más servicios de atención prenatal y ginecológica que la local. Además, se ha observado un claro gradiente socioeconómico en la mayoría de estas prestaciones.

El objetivo de este estudio fue comparar el uso efectivo de servicios de salud disponibles entre migrantes internacionales y chilenos. Dicha comparación permite identificar brechas de desigualdad en uso de servicios, que son potencialmente modificables, que pueden impactar negativamente en la salud de los migrantes y que van en contra de la meta universal de protección social en salud.

## MÉTODOS

Estudio observacional de corte transversal. Análisis secundario de encuesta de Caracterización Socioeconómica Nacional (CASEN), versión 2017^16^ . Esta encuesta permite observar patrones generales de población que se autorreporta como inmigrante (nacida en el extranjero) y compararla con la chilena (nacida en Chile).

La encuesta CASEN es desarrollada periódicamente por el Ministerio de Desarrollo Social. Es un instrumento de diagnóstico, evaluación y focalización, con el objetivo de conocer condiciones socioeconómicas de los hogares del país, especialmente aquellos grupos definidos como prioritarios por las políticas sociales. Esta encuesta, anónima y de libre disposición, utiliza un muestreo probabilístico de naturaleza compleja, permitiendo la representatividad de residentes en viviendas particulares de 324 comunas de las 16 regiones del país, excluyendo comunas de difícil acceso y personas institucionalizadas^16^ .

CASEN contó con 70.948 hogares en 2017, considerando una tasa de no respuesta del 26,6%. Esta encuesta contiene información de 216.439 personas residentes en hogares particulares, de las cuales 207.603 (representativos de 16.843.471) eran chilenas y 6.811 (representativas de 777.407) inmigrantes. Así, el 94,6% de la población representada era chilena y 4,4% inmigrante; 0,94% de los individuos no reportaron su estatus migratorio, y por ende fueron excluidos del análisis. La recolección de datos se realizó mediante entrevista estructurada directa a residentes habituales o un adulto de la familia, teniendo la facultad de responder por otros miembros del hogar.

Las variables del estudio fueron:

1.Acceso al sistema de salud

- Pertenencia a un sistema de previsional de salud [sí/no].
- Tipo de previsión de salud [pública/privada/otra].

Ambos creados a partir de la recodificación de la pregunta “¿A qué sistema previsional de salud pertenece usted? [1. Sistema Público FONASA grupo A, 2. Sistema Público, FONASA grupo B,3. Sistema Público FONASA grupo C, 4. Sistema Público, FONASA grupo D, 5. Sistema Público FONASA no sabe Grupo, 6. FF.AA. y del Orden, 7. ISAPRE, 8. Ninguno (particular), 9. Otro sistema]”

2.Indicadores de uso efectivo de servicios de salud^17^ : Autorreportes construidos a partir de las variables disponibles en encuesta CASEN 2017^16^ ( Figura 1 ).

**Figura 1:** Uso efectivo de servicios de salud para necesidades a corto y largo plazo: necesidad sentida, expresión de demanda, satisfacción de necesidad sentida, cobertura de Garantías Explícitas en Salud (AUGE-GES) y barreras de acceso a atención en salud. Encuesta de Caracterización Socioeconómica Nacional (CASEN), Chile, 2017.

- Necesidad sentida de salud: A corto plazo (¿tuvo algún problema de salud o accidente en los últimos tres meses? [sí/no]) y a largo plazo (Durante los últimos 12 meses ¿ha estado en tratamiento médico? [sí/no]).
- Demanda expresada y no expresada: Haber consultado o no al sistema de salud chileno, para aquellos que reportaron haber tenido alguna necesidad de salud de corto plazo [sí/no]. Y encontrarse cubierto o no el tratamiento por el sistema AUGE-GES en el caso de las necesidades a largo plazo.
- Demanda satisfecha y no satisfecha: (sí) Consulta o cobertura de la necesidad sentida en salud a corto o largo plazo, respectivamente, o no consulta o cobertura por motivos voluntarios. (no) no consulta o cobertura por motivos involuntarios.
- Razones de no consulta (¿Por qué no tuvo consulta ni atención?) o cobertura (¿Por qué este tratamiento médico no fue cubierto por el sistema AUGE-GES?).
- Barreras de atención: Presencia de problemas durante la consulta por necesidad a corto plazo [sí/no]. Indicador construido a partir de las preguntas: Cuando consultó, ¿se le presentó alguno de los siguientes problemas? a) llegar a la consulta, hospital, consultorio, etc. b) conseguir una cita/atención (hora). c) ser atendido en el establecimiento (demora en la atención, cambios de hora, etc.) d) pagar por la atención debido al costo e) entrega de medicamentos en el establecimiento de salud o acceso a ellos por su costo.

En los casos de necesidad sentida de salud a corto y largo plazo, un mayor valor del indicador reflejaba una peor condición de salud. Por otro lado, un mayor valor en los indicadores de: no pertenencia a un sistema previsional de salud, demanda no expresada, no consulta, no cobertura, no consulta o no cobertura por motivos involuntarios, necesidad no satisfecha y presencia de barreras de acceso, indicaba un peor acceso a servicios de salud.

3.Variables sociodemográficas de control: edad, sexo, zona de residencia [urbano/rural], nivel educacional [sin educación/primaria/secundaria/técnica o profesional], condición de actividad [ocupados/desocupados/inactivos], quintil autónomo del ingreso total del hogar.

Los indicadores de acceso al sistema de salud y uso efectivo de servicios de salud fueron analizados descriptivamente para inmigrantes y nacidos en Chile, en su totalidad y estratificados según variables sociodemográficas. Se analizó la independencia entre la condición migratoria e indicadores de uso efectivo de servicios de salud y acceso al sistema de salud mediante el test de Pearson con corrección de segundo orden Rao y Scott (estadístico F).

Se realizaron modelos de regresión logística, ajustados por variables sociodemográficas, considerando Pertenencia a un sistema de previsional de salud, Demanda satisfecha a corto y largo plazo y Barreras de atención, como variables dependientes, y condición de inmigrante como variable independiente. Esto para el total de la población y para mayores de 14 años (adultos) considerando adicionalmente la variable condición de actividad.

El manejo de base de datos y análisis de estos se realizó mediante el software STATA 14 (StataCorp), considerando el diseño complejo de la encuesta (estrato conglomerado y factor de expansión) y una estimación de varianza por linealización por series de Taylor. Se consideró una significancia de 0,05 y confianza del 95% para todos los análisis.

## RESULTADOS

Un 16,3% de los inmigrantes del país afirmaban no pertenecer a un sistema previsional de salud, valor siete veces mayor en comparación con los nacidos en Chile (2,3%).

Los inmigrantes presentaron significativamente una menor necesidad sentida en salud que los nacidos en Chile tanto a largo como a corto plazo. A corto plazo, en inmigrantes, un 15,1% del total de la población reportó enfermedad o accidente en los tres meses previos a la encuesta (vs 20,2% en chilenos); de estos, el 9,3% no consultaron y 1,7 de cada 100 no logró satisfacer su necesidad en salud al no consultar por motivos ajenos a su voluntad. En chilenos, estos valores corresponderán a 6,1% y 0,7 de cada 100, respectivamente, significativamente menores que en inmigrantes. Por el contrario, inmigrantes y chilenos reportaron una proporción similar de presencia de problemas durante la consulta (barreras de acceso): aproximadamente un 25,0% del total de consultantes por necesidades a corto plazo (25.179 inmigrantes, 774.792 chilenos). A largo plazo, un 9,6% de los inmigrantes se encontraba en tratamiento médico por alguna enfermedad durante el año previo a la encuesta (vs 26,2% en chilenos), de los cuales el 44,4% eran por otra condición de salud distinta a las 20 principales patologías AUGE-GES consultadas; este porcentaje correspondió al 27,2% en chilenos. Los inmigrantes reportaron significativamente una mayor proporción de no cobertura (44,6% inmigrantes, 15,2% chilenos) y necesidades no satisfechas (15,0% inmigrantes, 3,2% chilenos), siendo específicamente 2,9 y 4,7 veces mayor en inmigrantes que en chilenos, respectivamente ( Tabla 1 ). En términos de razones de chance (OR), los resultados fueron consistentes con lo observado a nivel descriptivo. Sin embargo, se presentan algunas diferencias en la magnitud de las disparidades entre inmigrantes y locales tras ajustar por variables sociodemográficas.

**Tabla 1:** Acceso al sistema de salud, uso efectivo de servicios de salud por necesidades sentida a corto y largo plazo y barreras de acceso a atención en salud en inmigrantes y nacidos en Chile. Encuesta de Caracterización Socioeconómica Nacional (CASEN), Chile, 2017

	Inmigrante		Nacido en Chile		Razón Inmigrante: Chileno	
	
	Cantidad	Indicador	Cantidad	Indicador		
**Sin previsión de salud**	123.013	**16,3**	378.239	**2,3**	7,1	*

**Necesidad sentida (Corto plazo)**	116.187	**15,1**	3.361.433	**20,2**	0,8	*
**Demanda no expresada**	10.720	**9,3**	203.530	**6,1**	1,5	*
Motivos de no consulta:						
Voluntaria	8.042	**80,9**	158.604	**87,1**	0,9	
Involuntaria	1.904	**19,1**	23.449	**12,9**	1,5	
**Necesidad no satisfecha**	1.904	**1,7**	23.449	**0,7**	2,4	*
**Barreras de acceso**	25.179	**24,7**	774.792	**25,4**	1,0	
Problemas para:						
Llegar a la consulta	4.105	**4,0**	218.147	**7,1**	0,6	*
Conseguir una cita/atención	14.724	**14,4**	390.782	**12,8**	1,1	
Ser atendido en el establecimiento	13.186	**13,0**	501.580	**16,4**	0,8	
Pagar por la atención	5.585	**5,5**	171.711	**5,6**	1,0	
Entrega de medicamentos	7.403	**7,5**	227.843	**7,2**	1,0	

**Necesidad sentida (Largo plazo)**	74.216	**9,6**	4.374.959	**26,2**	0,4	*
**No cobertura**	16.878	**44,6**	450.398	**15,2**	2,9	*
Motivos de no consulta:						
Voluntaria	6.974	**44,2**	230.410	**55,3**	0,8	
Otra razón, no especificada	3.865	**24,5**	96.017	**23,0**	1,1	
Involuntaria	4.953	**31,4**	90.330	**21,7**	1,4	
**Necesidad no satisfecha**	4.953	**15,0**	90.330	**3,2**	4,7	*

* Indicador no independiente de la condición de inmigrante (α=0,05). Test F, corrección de segundo orden Rao y Scott.

Tras ajustar por variables sociodemográficas, los inmigrantes presentan 7,5 veces más chances de no tener previsión de salud que los chilenos. Este chance fue 6,2 veces mayor en inmigrantes que en chilenos en mayores de 15 años y considerando la condición de actividad (ocupados, desocupados e inactivos).

El sexo, el tener educación media o superior en comparación con no tener educación, y el pertenecer el quintil de ingreso más rico en comparación con el más pobre, resultaron ser factores significativos en la falta de previsión de salud, en desventaja de las mujeres, personas sin educación y de menores ingresos para el total de la población. En mayores de 15 años, adicionalmente, la edad y desocupación en referencia a la ocupación resultaran ser significativas. Por otro lado, el tener educación media en referencia a no tener educación dejó de ser significativa ( Tabla 2 ).

**Tabla 2:** Determinantes de la falta de previsión de salud, demanda no expresada por necesidades a corto plazo, necesidades no satisfechas a corto plazo, presencia de barreras de acceso durante la atención, no cobertura Garantías Explícitas en Salud (AUGE-GES) por necesidades a largo plazo y necesidades no satisfechas a largo plazo. Encuesta de Caracterización Socioeconómica Nacional(CASEN), Chile, 2017.

	Acceso al sistema de salud				

	Sin previsión de salud				

	OR	IC95%			
Modelo 1					
**Inmigrante**	**7,5**	**6,08**	**-**	**9,29**	*
**Chileno**	**1**				
Rural	1,1	0,92	-	1,21	
Urbano	1				
Mujer	0,6	0,56	-	0,66	*
Hombre	1				
Edad	1,0	0,99	-	1,00	
Básica	1,2	0,83	-	1,70	
Media	1,8	1,25	-	2,69	*
Superior	2,6	1,82	-	3,64	*
Sin educación	1				
Quintil II	1,1	0,92	-	1,23	
Quintil III	1,0	0,91	-	1,22	
Quintil IV	1,0	0,78	-	1,29	
Quintil V (más rico)	0,6	0,50	-	0,78	*
Quintil I (más pobre)	1				
cte.	0,0	0,02	-	0,03	*

Modelo 2					
**Inmigrante**	**6,2**	**5,10**	**-**	**7,43**	*
**Chileno**	**1**				
Rural	1,0	0,91	-	1,20	
Urbano	1				
Mujer	0,6	0,52	-	0,64	*
Hombre	1				
Edad	1,0	0,98	-	0,99	*
Básica	1,2	0,89	-	1,68	
Media	1,3	0,93	-	1,79	
Superior	1,7	1,20	-	2,38	*
Sin educación	1				
Quintil II	1,1	0,93	-	1,24	
Quintil III	1,1	0,97	-	1,31	
Quintil IV	1,1	0,92	-	1,33	
Quintil V (más rico)	0,8	0,62	-	0,98	*
Quintil I (más pobre)	1				
Desocupados	2,9	2,48	-	3,30	*
Inactivos	1,0	0,90	-	1,11	
Ocupados	1				
cte.	0,0	0,03	-	0,05	*

*Significativo, con una significancia 0,05

Modelo 1: N=17.177.754 Df=1.295 F=96,05

Modelo 2: N=13.857.106 (15 año o más) Df=1.295 F=83,76

Dentro de los 632.770 inmigrantes con previsión de salud, el 80,0% pertenecía al sistema público, el 18,0% al privado y el 2,0% fuerzas armadas (FF.AA.) u otro, mientras que en chilenos estos porcentajes fueron 82,1%, 15,0% y 2,9% respectivamente. La proporción de pertenecientes al sistema privado de salud en hombres que en mujeres fue mayor tanto en inmigrantes como en nacidos en Chile. El 15,2% (IC95%:12,26–18,79%) de las mujeres con previsión pertenecían al sistema público, mientras que en hombres un 21,0% (IC95%:16,78–26,03%) en inmigrantes. [Resultados no presentados en tabla]

La población inmigrante presentó significativamente 21,0% (OR = 0,79) menos chance de tener necesidades a corto plazo en salud (enfermedades o accidentes) que los chilenos. Sin embargo, tuvieron 1,7 veces más chance de no consultar ante esta necesidad y 3,1 veces más chance de no satisfacer esta, es decir, no haber consultado por motivos involuntarios. Todo esto tras ajustar por variables sociodemográficas. Esta situación se mantuvo en mayores de 15 años. Sexo, edad, educación y pertenecer al quintil más rico en contraste con el más pobre fueron variables significativas en la no expresión de la demanda. La educación dejó de ser significativa en mayores de 15 años, y el resto de los quintiles de ingreso (a excepción del III) y la condición de actividad pasaron a serlo ( Tabla 3 ).

**Tabla 3:** Determinantes de la demanda no expresada por necesidades a corto plazo, necesidades no satisfechas a corto y largo plazo, presencia de barreras de acceso durante la atención y no cobertura Garantías Explícitas en Salud (AUGE-GES). Encuesta de Caracterización Socioeconómica Nacional (CASEN),, Chile,2017.

	Corto Plazo									Largo Plazo					
	
	Demanda no expresada^a^			Necesidad no satisfecha^b^			Barreras de acceso^c^			No cobertura AUGE–GES^a^			Necesidad no satisfecha^b^		
				
	OR	IC95%		OR	IC95%		OR	IC95%		OR	IC95%		OR	IC95%	
**Inmigrante**	**1,7**	**1,22–2,47**	*	**3,1**	**1,31–7,44**	*	**1,2**	**0,75–1,84**		**2,7**	**1,97–3,73**	*	**3,3**	**1,89–5,74**	*
**Chileno**	**1**			**1**			**1**			**1**					
Rural	1,2	0,94–1,43		1,2	0,71–1,88		1,0	0,86–1,18		0,6	0,50–0,68	*	0,6	0,46–0,86	*
Urbano	1			1			1			1					
Mujer	0,8	0,72–0,88	*	0,9	0,66–1,28		1,0	0,96–1,08		1,0	0,88–1,04		1,0	0,89–1,23	
Hombre	1			1			1			1					
Edad	1,0	1,00–1,01	*	1,0	1,01–1,02	*	1,0	1,00–1,01		1,0	0,98–0,99	*	1,0	0,98–0,99	*
Básica	1,5	1,14–1,92	*	1,8	0,83–4,08		1,0	0,90–1,13		1,0	0,83–1,33		0,9	0,61–1,26	
Media	1,8	1,38–2,25	*	2,3	1,08–4,92	*	1,0	0,87–1,11		1,7	1,35–2,15	*	1,2	0,83–1,73	
Superior	1,8	1,39–2,35	*	2,0	0,86–4,62		0,9	0,80–1,04		3,4	2,63–4,29	*	2,0	1,40–3,00	*
Sin educación	1			1			1			1					
Quintil II	0,9	0,74–1,11		0,8	0,47–1,22		0,9	0,81–0,99	*	1,3	1,08–1,53	*	1	0,75–1,35	
Quintil III	1,0	0,78–1,18		0,7	0,41–1,06		0,8	0,69–0,90	*	1,8	1,52–2,08	*	1,6	1,19–2,26	*
Quintil IV	0,9	0,69–1,08		0,9	0,47–1,93		0,7	0,58–0,74	*	2,7	2,30–3,22	*	1,9	1,43–2,63	*
Quintil V (más rico)	0,6	0,44–0,74	*	0,3	0,14–0,69	*	0,4	0,35–0,50	*	5,5	4,57–6,53	*	2,3	1,69–3,27	*
Quintil I (más pobre)	1			1			1			1					
cte.	0,0	0,03–0,05	*	0,0	0,00–0,00	*	0,4	0,32–0,43	*	0,1	0,09–0,16	*	0,0	0,03–0,07	*

**Inmigrante**	**1,7**	**1,18–2,40**	*	**2,89**	**1,17–7,13**	*	**1,2**	**0,77–1,88**		**2,8**	**2,01–3,85**	*	**3,3**	**1,87–5,78**	*
**Chileno**	**1**			**1**			**1**			**1**			**1**		
Rural	1,1	0,89–1,27		1,1	0,64–1,75		1,0	0,83–1,13		0,6	0,52–0,70	*	0,6	0,47–0,89	*
Urbano	1			1			1			1			1		
Mujer	0,8	0,72–0,89	*	1,0	0,71–1,52		1,0	0,98–1,13		1,0	0,88–1,06		1,1	0,93–1,31	
Hombre	1			1			1			1			1		
Edad	1	1,00–1,01	*	1,0	1,00–1,02		1	1,00–1,01		1,0	0,99–0,99	*	1,0	0,98–0,99	*
Básica	1,0	0,71–1,35		1,2	0,46–2,90		1,1	0,92–1,36		1,1	0,84–1,54		0,8	0,51–1,24	
Media	1,0	0,74–1,40		1,1	0,44–2,70		1	0,81–1,23		2,0	1,51–2,79	*	1,1	0,70–1,85	
Superior	1,0	0,72–1,37		0,9	0,32–2,41		0,9	0,71–1,11		4,2	3,05–5,71	*	1,9	1,18–3,18	*
Sin educación	1			1			1			1			1		
Quintil II	0,8	0,66–0,98	*	0,7	0,42–1,10		0,9	0,82–1,02		1,2	1,05–1,47	*	1,0	0,72–1,34	
Quintil III	0,8	0,69–1,04		0,6	0,37–0,96	*	0,8	0,71–0,95	*	1,7	1,47–2,04	*	1,6	1,18–2,31	*
Quintil IV	0,7	0,59–0,92	*	0,8	0,40–1,48		0,7	0,64–0,82	*	2,5	2,13–3,02	*	1,9	1,39–2,67	*
Quintil V (más rico)	0,5	0,39–0,68	*	0,3	0,12–0,62	*	0,5	0,39–0,57	*	5,0	4,20–6,08	*	2,43	1,71–3,44	*
Quintil I (más pobre)	1			1			1			1			1		
Desocupados	0,7	0,47–0,93	*	0,8	0,39–1,78		1,6	1,17–2,09	*	1,4	1,06–1,76	*	2,1	1,41–3,01	*
Inactivos	0,8	0,67–0,86	*	0,5	0,30–0,76	*	1,0	0,95–1,11		1	0,89–1,11		1	0,81–1,23	
Ocupados	1			1			1			1			1		
cte.	0,1	0,07–0,16	*	0,0	0,00–0,02	*	0,3	0,27–0,46	*	0,1	0,05–0,12	*	0,0	0,02–0,07	*

*Significativo, con una significancia 0,05

AUGE –GES: Sistema de Garantías Explicitas en Salud

De manera cruda, los inmigrantes tenían 2,0% (OR = 0,98) veces menos chance de presentar barreras de acceso que los chilenos. Tras ajustar por variables sociodemográficas, esta situación se invirtió. Los inmigrantes presentaran una mayor chance de presentar barreras de acceso tanto para el total de la población como para los de 15 años o más. Para ninguno de los casos esto fue significativo ( Tabla 3 ).

Para las necesidades a largo plazo (estar en tratamiento por alguna enfermedad) de manera significativa, los inmigrantes tuvieron un mayor chance de no tener su tratamiento cubierto pese a presentar un menor chance de estar en tratamiento. Específicamente, ajustando por variables sociodemográficas, los inmigrantes tenían 53,0% (OR = 0,47) veces menos chance de estar en tratamiento por alguna enfermedad que los chilenos y 37,0% (OR=0,63) veces menos chance de que esta enfermedad fuera una patología AUGE-GES. Entre aquellos con patologías AUGE-GES, la población inmigrante tenía 2,7 veces más chance de no estar cubierta y 3,3 veces más chance de no satisfacer esta necesidad de cobertura que los chilenos. En mayores de 15 años, los inmigrantes tuvieron mayor chance de no estar cubiertos por el AUGE-GES y no satisfacer su necesidad a largo plazo que los chilenos. A excepción del sexo y el tener educación básica en contraste con el no tener educación, todas las variables consideradas fueron significativas en la no cobertura y no satisfacción. En mayores de 15 años tampoco fue significativo el tener educación media en contraste con no tener educación ( Tabla 3 ).

Al analizar los modelos de indicadores de uso efectivo de servicios de salud estratificados por con y sin previsión de salud, la presencia de necesidades a largo plazo y cobertura de esta la variable condición de inmigrante fue significativa ( Figura 2 ).

**Figura 2:** Disparidad relativa de los inmigrantes en referencia a los chilenos en acceso al sistema de salud y uso efectivo de servicios de salud por necesidades a corto y largo plazo, expresada en Razón de chances (OR) ajustadas por variables sociodemográficas, según previsión de salud. Encuesta de Caracterización Socioeconómica Nacional (CASEN), Chile, 2017.

## DISCUSIÓN

Los resultados de este estudio indican que los inmigrantes aún se encuentran en desventaja en términos de acceso y uso a servicios de salud en comparación con los nacidos en Chile. Los inmigrantes presentaron 7,5 veces más chances de no tener previsión de salud que los chilenos y una menor necesidad sentida pero una menor consulta, cobertura o satisfacción de estas necesidades a corto y a largo plazo. Los inmigrantes tenían 1,7 veces más chance de no consultar ante una enfermedad o accidente y 3,1 veces más chance de no satisfacer su necesidad, al no consultar por motivos ajenos a su voluntad, que los nacidos en Chile. Por otro lado, la población inmigrante tenía 2,7 veces más chance de no tener cubierto su tratamiento por el AUGE-GES y 3,3 veces más chance de no satisfacer esta necesidad de cobertura que los chilenos.

Muchos países se han visto desafiados por el aumento global de población migrante internacional en los últimos años. Esto trae consigo una seria de desafíos para diversos sectores, educación, salud y protección social, solo por nombrar algunos, los cuales se vuelven más complejos de enfrentar cuando esta población se encuentra en condiciones de vulnerabilidad social. La protección de la salud en migrantes internacionales es de preocupación global, y el acceso a servicios de salud una arista fundamental para esta labor^17^ .

El porcentaje de inmigrantes de primera generación presentes en el país aumentó en 0,7% en Chile, entre el 2013 y 2015, lo cual se traduce en 110.738 inmigrantes^18^ . Por otro lado, se ha reportado que una proporción significativa de estos viven en condiciones de vulnerabilidad social, ya sea por encontrarse en situación irregular, pobreza, malas condiciones de vivienda, desempleo o empleo informal y procesos de discriminación y abuso o la dificultada para obtener documentación oficial de identidad de residencia en el país, lo cual en temas de acceso a salud es esencial para poder pertenecer al sistema previsional de salud^19^ . Para el año 2015, un 15,7% de los inmigrantes no se encontraba afiliado a ningún sistema de salud, valor 5,8 veces mayor que en nacidos en Chile. La población inmigrante se encontraba en situación de desventaja respecto a la población local también en otros indicadores de acceso a salud, como tasa de atención médica ante problema de salud y problema de acceso a atención^18^ .

Se observan las mismas falencias que en años anteriores por Cabieses y cols. en cuanto al uso efectivo de servicios de salud por necesidades a corto y largo plazo y acceso al sistema de salud^17^ . Más preocupante aun es que algunos de estos indicadores han aumentado entre 2013 y 2017. Un 15,6% de los inmigrantes no tenían previsión de salud en 2013, mientras que fueron un 16,3% en 2017; un 8,9% de los que tenían necesidades a corto plazo no consultaron por esta necesidad en 2013, mientras que fue un 9,4% en 2017. Un 27,9% de los que se encontraban en tratamiento por una enfermedad AUGE-GES no se encontraban cubiertos por el mismo en 2013, mientras que este valor fue de 44,6% en 2017.

Es necesario reducir brechas de desigualdad en acceso y uso de servicios de salud por parte de población migrante. Para eso, son necesarias estrategias concretas en salud y políticas que permitan, a través los determinantes sociales de la salud, proteger la salud y bienestar de migrantes, desde la aparición de problemas de salud de corto y largo plazo, y factores que puedan favorecer estos, hasta su efectiva resolución. Intervenciones que consideren un enfoque de participación social de la comunidad migrante internacional y que acerquen el sistema de salud chileno a esta población son ampliamente recomendadas^17^ ; más aún en Chile, en donde según la encuesta CASEN (2015), un 18,5% de los inmigrantes de 12 años o más han participado o participan en alguna organización o grupo organizado^18^ . La encuesta CASEN en todas sus versiones es de representación nacional y permite obtener un diagnóstico de la situación de la población inmigrante en el país respecto a diversas condiciones socioeconómicas. En este caso específico de la salud, esta permite conocer: condiciones de salud (necesidades, estado general de salud y condiciones permanentes o de larga duración), acceso al sistema, servicios de salud, seguros de salud, uso de estos y pagos asociados, las cuales a su vez pueden asociarse entre ellas y entre una amplia diversidad de factores demográficos, culturales y socioeconómicos^17^ . Pese a esto, el proceso de selección muestral de la encuesta no busca representar a la población migrante internacional en particular y la variable de país de nacimiento (que permite la estratificación entre inmigrante y chileno) cuenta con porcentajes no respuesta (responde “no sabe”) en 2017 del 0,9%. Por otro lado, la encuesta CASEN solo permite el análisis de residentes en viviendas, dejando fuera del panorama a aquellos inmigrantes en situación de calle, los cuales según medios locales se encuentran en incremento^20^ . Los análisis realizados a partir de esta encuesta se encuentran limitados a las variables presentes en ella. Algunos de los desafíos culturales como diferencias en visiones, tradiciones y estilos de vida, entre otros, que se traducen en barreras de integración y por ende en barreras y desafíos en el acceso a sistemas y servicios de salud^19^ no pueden ser analizados directamente. Es, por tanto, de amplia utilidad a futuro complementar los análisis presentados en este manuscrito.

El presente estudio indica que persisten las desventajas en acceso y uso a servicios de salud en inmigrantes en comparación con los nacidos en Chile. Es necesario reducir las brechas entre inmigrantes y nacidos en Chile, sobre todo en cuanto a pertenencia a un sistema de salud. Esta es la primera barrera para un uso efectivo de servicios. Se sugiere generar estrategias concretas y políticas en salud que consideren un enfoque de participación social de la comunidad inmigrante y, adicionalmente, acerquen al sistema de salud a esta población.

Modelo Demanda (corto plazo): N=3.415.253Df=1.289 F=10,64. Modelo Satisfacción necesidad (corto plazo): N=3.393.049 Df=1.289 F=6,91. Modelo Barreras: N=3.146. Df=1.288 F=17,78. Modelo Demanda en adultos (corto plazo): N=2.797.755 Df=1.288 F=6,54. F=6,08. Modelo Satisfacción necesidad en adultos (corto plazo): N=2.778.419 Df=1.288 F=3,72. Modelo barreras en adultos: N=2.563.952 Df=1.287 F=13,76. Modelo Cobertura: N=2.974.923 Df=1.295 F=118,55. Modelo Cobertura en adultos: N=2.840.844Df=1.295 F=25,94. Modelo Satisfacción necesidad (largo plazo): N=2.853.809 Df=1.295 F=100,76. Modelo Satisfacción necesidad en adultos (largo plazo): N=2.727.317 Df=1.295 F=21,8
